# An Acoustofluidic Device for Sample Preparation and Detection of Small Extracellular Vesicles

**DOI:** 10.34133/cbsystems.0319

**Published:** 2025-07-17

**Authors:** Jessica F. Liu, Jianping Xia, Joseph Rich, Shuaiguo Zhao, Kaichun Yang, Brandon Lu, Ying Chen, Tiffany Wen Ye, Tony Jun Huang

**Affiliations:** ^1^ Department of Anesthesiology, Duke University School of Medicine, Durham, NC 27710, USA.; ^2^ The Thomas Lord Department of Mechanical Engineering and Materials, Duke University, NC 27708, USA.; ^3^ Department of Biomedical Engineering, Duke University, Durham, NC 27708, USA.

## Abstract

Small extracellular vesicles (sEVs) have emerged as powerful vectors for liquid biopsy, offering a noninvasive window into the dynamic physiological and pathological states of the body. However, to fully leverage the clinical potential of sEV biomarkers, it is imperative to develop robust and efficient technologies for their isolation and analysis. In this study, we introduce a novel sharp-edge acoustofluidic platform designed for rapid and effective sample preparation, coupled with sensitive detection of specific sEV populations based on their surface markers. Our approach utilizes acoustically activated sharp-edge microstructures to concentrate bead-bound sEVs within the microfluidic device, facilitating immediate visualization by fluorescence microscopy. As a proof of principle, we demonstrate the capability of this portable acoustofluidic chip to selectively isolate and detect epidermal growth factor receptor (EGFR)-expressing vesicles, achieving nearly a 6-fold signal enhancement in EGFR-positive sEVs compared to EGFR-negative populations from sample volumes as small as 50 μl. This advancement not only underscores the potential of our platform for high-sensitivity biomarker detection but also paves the way for its application in isolating organ-specific sEVs. Such capability could be transformative for real-time monitoring of organ function and the simultaneous detection of multiple sEV markers, thereby broadening the scope of diagnostic precision and therapeutic decision-making in clinical practice.

## Introduction

Nano-sized vesicles (30 to 140 nm), known as small extracellular vesicles (sEVs), are released by all cells in the body [1]. These vesicles are present in various biofluids, including peripheral blood, urine, saliva, and cerebrospinal fluid. Once considered cellular waste, they are now recognized as crucial mediators of intercellular communication and valuable indicators of organ function. sEVs contain distinct genomic, proteomic, and metabolomic markers, providing deep insights into cellular processes and playing key roles in cell signaling, inflammation, and cancer progression [2,3]. As a result, sEVs have emerged as a powerful vector for liquid biopsy, enabling the noninvasive assessment of physiological and pathological states [4,5].

However, clinical applications of sEV analysis are often constrained by the limited availability of patient-derived samples, necessitating highly efficient processing and detection methods [6,7]. Conventional sEV processing techniques struggle with rapid and reliable detection in small sample volumes, presenting a major hurdle for point-of-care diagnostics [8,9]. Nano flow cytometry enables high-throughput sEV analysis by detecting specific markers and sorting vesicle populations [10]. However, this approach requires bulky, expensive instrumentation and skilled operators, often necessitating additional preprocessing steps [11,12]. Similarly, techniques such as Western blotting involve lengthy, complex protocols and typically do not allow intact sEV subpopulations to be recovered for downstream analysis [13–16].

Microfluidic technologies offer a transformative solution for the rapid and efficient processing of sEVs [17–21]. These platforms allow the manipulation of small biofluid volumes, drastically reducing sample requirements and processing time while integrating multiple functions—such as isolation, purification, and detection—on a single compact chip. Notably, on-chip immunoaffinity capture and electrochemical detection have demonstrated high sensitivity and specificity in identifying distinct sEV populations [22].

In this work, we introduce an acoustofluidic [23–39] platform that seamlessly integrates acoustics and microfluidics for sEV sample preparation and detection. Our device consists of a single PDMS microchannel embedded with sharp-edge microstructures, which are activated by an acoustic buzzer. Upon activation, these structures generate fluid vortices that selectively concentrate microparticles (>1 μm) at the tips of the microstructures while allowing nanoparticles (<400 nm) to remain distributed. By leveraging antibody-coated micrometer-sized beads to “tag” sEVs, we achieve highly specific concentration and detection at the microstructure tips using fluorescence microscopy.

We validate our acoustofluidic platform by successfully capturing and detecting epidermal growth factor receptor (EGFR)-expressing sEVs, achieving a nearly 6-fold signal enhancement for EGFR-positive sEVs compared to EGFR-negative controls. The versatility of this platform allows for straightforward adaptation to other sEV markers simply by changing the antibody tag. Critically, the acoustic waves [40] employed in our system operate at biologically safe frequencies, ensuring sample integrity, while the device itself is powered by conventional benchtop electronics, making it highly accessible. Unlike traditional technologies, our acoustofluidic device uniquely combines rapid sample preparation, high detection accuracy, portability, and the ability to process ultra-small volumes (as little as 50 μl)—addressing a critical gap in current sEV sample preparation and analysis methods. This innovation paves the way for a new generation of cost-effective, user-friendly diagnostic tools that will revolutionize sEV-based liquid biopsies and point-of-care disease monitoring.

## Methods

### Device fabrication

The acoustofluidic device comprises a polydimethylsiloxane (PDMS) layer with a microchannel containing sharp-edge microstructures on a glass substrate to which an acoustic wave generator is adhered. The fluid channel with sharp-edge structures was fabricated using soft lithography techniques [41]. Briefly, PDMS (Ellsworth) was molded using an SU-8/silicon wafer pattern to form a 100-μm-tall × 800-μm-wide microchannel containing 400-μm-long sharp-edge microstructures spaced at 400-μm intervals on alternating sides of the channel. The channel was then plasma bonded onto a 25 mm × 50 mm glass slide (Fisher) to form the base of the channel. Finally, an acoustic buzzer (Digikey, No. 668-1407-ND) capable of activating the sharp-edge microstructures was adhered to the glass slide at one end of the microchannel using clear epoxy (Amazon).

### Simulation

To calculate the acoustic radiation force and drag force within the microfluidic channel, simulations were performed using COMSOL Multiphysics. In particular, a customized 3-dimensional model was developed based on the actual geometry parameters of the microfluidic channel. The Solid Mechanics, Thermoviscous Acoustics, and Electrostatic modules were coupled using the built-in multiphysics interface in COMSOL to simulate the transducer displacement, acoustic pressure distribution, and streaming velocity field within the microfluidic channel. A voltage terminal and ground terminal were applied to opposite sides of the buzzer to generate acoustic waves, and the model was solved using a frequency-domain study.

Following the calculation of the acoustic pressure and velocity fields, the Acoustic Streaming was determined using the Acoustic Streaming domain coupling interface, solved with a steady-state solver. The acoustic radiation force was derived from the Gor’kov potential, defined as:

$$U=V_{p}\left(\frac{f_{1}}{2\rho_{0}{c_{0}}^{2}}<p\cdot p^{*}>-\frac{3}{4}\rho_{0}\;f_{2}<v\cdot v^{*}>\right)$$ (1)

where:

$$f_{1}=1-\frac{\rho_{0}{c_{0}}^{2}}{\rho_{p}{c_{p}}^{2}}$$ (2)

$$f_{2}=\frac{2\left(\rho_{p}-\rho_{0}\right)}{2\rho_{p}+\rho_{0}}\;$$ (3)

Here, *ρ_0_* = 1,000 kg/m^3^ and *c_0_* = 1,500 m/s are the density and sound speed of water, and *ρ*_p_ = 1,020 kg/m^3^ and *c*_p_ = 2,320 m/s are the density and sound speed of the particles. *V*_p_ is the particle volume, calculated for a particle with a diameter of 5 μm, and < **·** > represents the time-averaging operator.

The drag force was calculated using the expression:

$$F_{\text{drag}}=6\pi \mu R\left(v_{s}-v_{p}\right)$$ (4)

where *μ* is the dynamic viscosity of water, *R* is the particle radius (2.5 μm), *v*_s_ is the acoustic streaming velocity, and *v*_p_ is the particle velocity.

### Extracellular vesicle labeling

To demonstrate proof-of-concept application of the device, fluorescent sEVs were required. To obtain nonspecific fluorescent sEVs, samples were obtained from cell culture media using ExoQuick-TC precipitation solution (System Biosciences). Briefly, the cell culture medium was centrifuged at 3,000*g* × 15 min to remove cells and large debris. The supernatant was then mixed with ExoQuick-TC reagent at a 5:1 v/v ratio and incubated at 4 °C overnight. After incubation, the mixture was centrifuged at 1,500*g* × 30 min. The supernatant was carefully removed, and the pellet was again centrifuged at 1,500*g* × 5 min to remove any remaining media. The pellet was resuspended in 1× phosphate-buffered saline (PBS).

sEVs were fluorescently labeled using a PKH67 kit (Sigma-Aldrich). Briefly, Diluent C from the kit was added to the sEV suspension to a volume of 1 ml. Next, 6 μl of PKH67 dye was added to the sample. After 5 min at room temperature, the reaction was quenched with 2 ml of 10% bovine serum albumin (BSA) in PBS. To remove excess dye, the fluorescently stained sEVs were then re-mixed with a 5:1 v/v ratio of ExoQuick (System Biosciences), incubated at 4 °C × 1 h, and processed as described above. Finally, sEV size was characterized by dynamic light scattering using a Zetasizer (Malvern).

### Separation of microparticles from nanoparticles

To demonstrate separation of microparticles from nanoparticles, *d* = 5 μm fluorescent red Nile polystyrene beads (Spherotech) were mixed with *d* = 400 nm green fluorescent polystyrene nanoparticles (Spherotech) and loaded into the device. A square wave was applied to the acoustic buzzer to activate acoustic vortexing at the sharp-edge structures within the device. Fluorescence images were acquired by microscopy (Olympus).

Similarly, to demonstrate separation of microparticles from sEVs, *d* = 5 μm fluorescent red Nile polystyrene beads (Spherotech) were mixed with fluorescently stained sEVs and loaded into the sharp-edge acoustofluidic device. A square wave was applied to the acoustic buzzer to activate acoustic vortexing at the sharp-edge structures within the device. Fluorescence images were acquired by microscopy (Olympus).

### Streptavidin-coated bead and biotin-coated nanoparticle binding

Streptavidin-coated polystyrene beads (5 μm, Nanocs) at various concentrations were mixed with 200-nm biotin-coated green fluorescent latex nanoparticles (ThermoFisher) at a concentration of 2 × 10^8^ μl^−1^. The effectiveness of particle aggregation was validated by comparing different bead sizes, showing that 5-μm beads formed more stable aggregates under acoustic excitation than 2-μm beads (Fig. S5). After 15 min, the sample was loaded onto the device and a square wave was applied to the acoustic buzzer. Fluorescent images were acquired by microscopy (Olympus).

To enable comparison of streptavidin bead–biotin nanoparticle aggregates from across different concentrations, a fluorescence intensity ratio (FIR) was calculated. Specifically, the fluorescence signal at the tip of the sharp-edge microstructures within a 75 μm × 75 μm region was normalized to the background fluorescence along the edge of the microchannel within a region of the same size.

### sEV capture using anti-CD63-coated beads

To demonstrate sEV detection using the acoustofluidic device, sEVs obtained from cell culture media were fluorescently stained and labeled with 4.5-μm anti-CD63-coated Dynabeads (ThermoFisher). Briefly, 2 × 10^5^ beads were washed with 200 μl of Isolation buffer (ThermoFisher) and incubated with fluorescently labeled sEVs at 4 °C × 12 h in PBS with 0.1% BSA. After incubation, the sample was loaded into the device, and a square wave was applied to the acoustic buzzer to activate the sharp-edge microstructures. Fluorescent images were acquired by microscopy (Olympus). A FIR was calculated as described above. Anti-mouse immunoglobulin G (IgG)-coated Dynabeads (ThermoFisher) were used as a negative binding control via the same protocol.

### Anti-EGFR bead synthesis

Anti-EGFR beads were synthesized by coupling EGFR polyclonal antibody (ThermoFisher) to M-280 Tosylactivated Dynabeads (ThermoFisher) according to the manufacturer’s protocol. Briefly, 5 mg of Dynabeads was washed with 1 ml of 0.1 M borate buffer, pH 9.5. The beads were combined with 100 μg of antibody in PBS and added to 100 μl of 3 M ammonium sulfate in 0.1 M borate buffer (pH 9.5). Following coupling at 37 °C for 18 h, the bead–antibody conjugates were washed with 1 ml of 0.5% BSA in PBS at 37 °C for 1 h. Finally, the beads were twice washed with 0.1% BSA in PBS and resuspended in the same.

### EGFR-specific sEV capture

To demonstrate the device’s ability to detect surface antigen-specific sEVs, sEVs isolated from the cell culture media of K562 (EGFR-negative) and HeLa (EGFR-positive) cells were fluorescently stained and labeled with antibody-coated beads. Briefly, 4.5-μm anti-EGFR-coated Dynabeads were washed with 200 μl of Isolation Buffer (ThermoFisher) and incubated with fluorescently labeled sEVs at 4 °C × 12 h in PBS with 0.1% BSA. After incubation, the sample was loaded into the device, and a square wave was applied to activate the sharp-edge microstructures. Fluorescent images were acquired by microscopy (Olympus), and a FIR was calculated as described above. Anti-CD63-coated Dynabeads (ThermoFisher) were used as a positive binding control, and anti-mouse IgG-coated Dynabeads (ThermoFisher) were used as a negative binding control via the same protocol.

### Image acquisition, processing, and data analysis

To standardize and optimize the fluorescent readout, images were acquired at the focal plane where the sharp-edge tips were optimally focused in the brightfield view. This corresponds to the mid-plane of the channel height, where acoustic streaming velocity is maximized due to the no-slip boundary condition at the top and bottom walls. To quantify signal enhancement, a FIR was calculated by comparing the average fluorescence intensity of a 75 μm × 75 μm region near a sharp-edge tip with that of a region of the same size near the edge of the microfluidic device. Each region contained multiple microparticles to ensure a representative signal, and measurements were averaged over the first 5 sharp-edge tips to reduce tip-to-tip variability. The FIR serves as a relative metric of particle aggregation. While using single-particle intensity as a baseline would provide a more rigorous assessment, such data were not acquired under matched imaging conditions in this study. Fluorescence microscopy images were processed using ImageJ software. Nested and unnested one-way analysis of variance (ANOVA) tests were performed in MATLAB to compare mean FIR values across experimental groups, and data are reported as mean ± standard error.

## Results

### Design of the acoustofluidic chip for specific sEV capture and detection

**Fig. 1.**
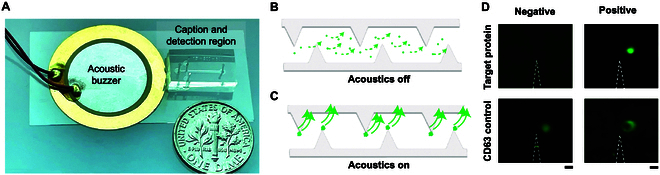
The acoustofluidic platform for capture and detection of sEVs via specific surface markers. (A) The acoustofluidic chip comprises a channel containing sharp-edge microstructures that can be acoustically activated with an acoustic buzzer. (B) In the absence of acoustic activation, no fluorescence signal is detected as sEVs pass through the channel in the detection region. (C) When the chip is acoustically activated, sEVs containing specific surface markers are concentrated at the tips of the sharp-edge microstructures and detectable by fluorescence microscopy. (D) Experimental demonstration of on-chip detection of sEVs based on their surface proteins. A fluorescent signal is observed when the target protein is present above the detection threshold, while no signal appears if the protein is absent. CD63 is used as a control biomarker due to its common presence on sEVs. Scale bar, 100 μm.

The acoustofluidic chip [42] we used in this work comprises a PDMS channel (sEV capture and detection region) on a glass substrate adjacent to an acoustic buzzer (Fig. 1A). The PDMS channel contains sharp-edge microstructures, which are activated by an acoustic field generated by the buzzer. In the absence of acoustic activation, sEVs are distributed throughout the channel in the detection region, and no fluorescence signal is seen (Fig. 1B). When the device is acoustically activated, bead-bound sEVs containing surface proteins [43] of interest are concentrated at the tips of the sharp-edge microstructures and generate a detectable fluorescence signal (Fig. 1C). Fig. 1D experimentally demonstrates this detection approach: Fluorescence appears only when the target protein is present above the detection threshold, while no signal is observed in its absence. CD63 serves as the control biomarker due to its widespread expression on sEVs, and fluorescence for CD63 is consistently observed regardless of the presence of the target surface protein in the sample.

### Acoustofluidic-enabled detection of large beads versus nanoparticles

**Fig. 2.**
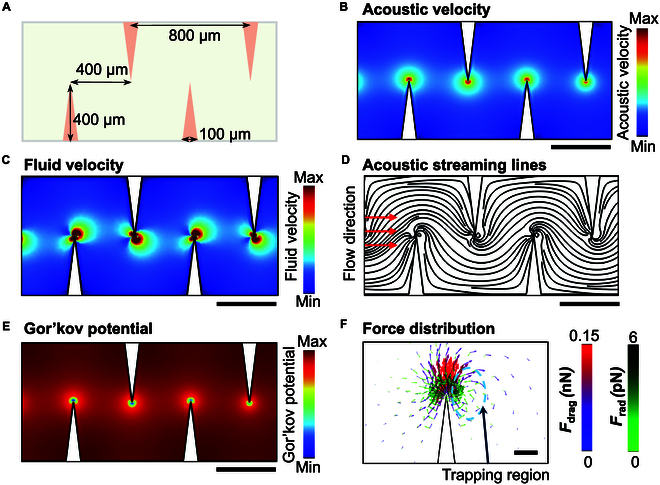
Mechanism of particle concentration at the tips of sharp-edge microstructures of the acoustofluidic device. (A) Geometry of the microfluidic channel and the sharp-edge structure in the acoustofluidic device. A detailed view of the structure is provided in the figure. (B) Simulated acoustic velocity field within the microfluidic channel, showing a maximum velocity at the tip of the sharp-edge structure. (C) Simulated acoustic streaming velocity field with an input background flow of 0.1 mm/s from left to right. (D) Simulated acoustic streaming lines, revealing a single vortex around the sharp edge. (E) Calculated Gor’kov potential distribution near the sharp-edge structure. Scale bars, 400 μm (B to E). (F) Distributions of the Gor’kov potential-induced acoustic radiation force (*F*_rad_) and acoustic streaming-induced drag force (*F*_drag_) around a sharp-edge structure. Scale bar, 20 μm.

The geometry of the sharp-edge structure was adapted from previous studies [44–49] and is illustrated in Fig. 2A. The height of the microchannel and sharp-edge structures is 50 μm, which was selected based on simulation results showing that taller channel heights reduce acoustic streaming velocity, while shorter channel heights increase the risk of channel blockage (Fig. S1). Elastic waves generated by the piezoelectric transducer propagate into the microfluidic channel, where their interaction with the sharp edges leads to a localized increase in acoustic velocity, peaking at the sharp-edge tip (Fig. 2B). The vibration of the acoustic field around the sharp edge generates acoustic streaming, with the streaming velocity also peaking at the sharp-edge tip.

The streaming patterns are visualized in Fig. 2C, where the streaming lines (Fig. 2D) reveal 2 vortices forming near each sharp-edge tip. Due to the presence of the background flow (0.1 mm/s), one of the vortices aligns with the flow direction, while the other opposes it. The opposing vortex is suppressed, resulting in a single dominant acoustic vortex around each sharp-edge tip. This acoustic streaming induces a strong drag force that guides particle motion along the streaming lines. However, since the drag force direction aligns with particle motion, it does not contribute to the centripetal force required for circular motion. Instead, the centripetal force is provided by the acoustic radiation force [50], which is directed toward the vortex center. The simulated acoustic streaming velocity fields and streamlines under varying background flow conditions are shown in Fig. S2. The Gor’kov potential distribution within the microfluidic channel is shown in Fig. 2E, illustrating the regions of stable particle trapping. The distributions of the drag force and acoustic radiation force around a sharp-edge tip are presented in Fig. 2F. As expected, the acoustic radiation force is directed toward the vortex center, acting as the centripetal force necessary for stable circular motion of the particles.

**Fig. 3.**
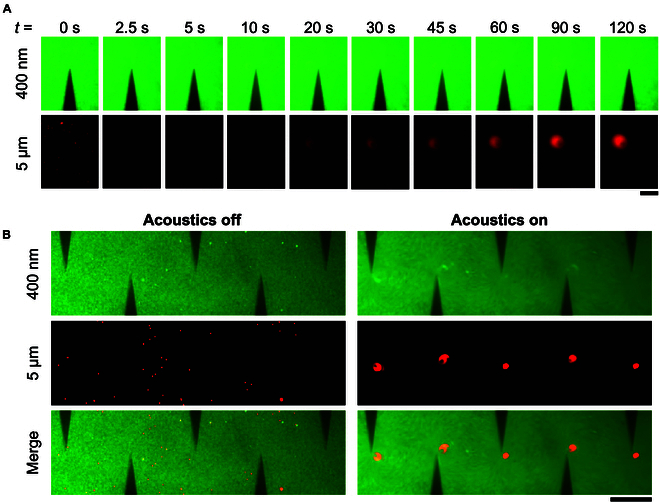
The acoustofluidic device demonstrates size-selective concentration of particles at the tips of sharp-edge microstructures. (A) Time series fluorescence imaging demonstrates that large 5-μm particles (red) rapidly accumulate at the tips of acoustically activated sharp-edge microstructures, while small 400-nm particles (green) remain distributed throughout the device. Scale bar, 100 μm. (B) This effect is seen throughout the sharp-edge microstructures in the device. Additionally, in the absence of acoustic activation, both large (red, 5 μm) and small (green, 400 nm) particles remain distributed throughout the device. Scale bar, 300 μm.

A series of acoustic vortices form around the sharp-edge, each with a low-velocity vortex center. Beads that capture sEVs with the target surface protein are trapped in these vortices to generate a fluorescent signal. To demonstrate that the acoustically activated device can be used to separate large microparticles from small nanoparticles, a sample containing 5-μm beads (red) and 400-nm nanoparticles (green) is loaded into the device. When a square wave is applied to the device, time series imaging with fluorescence microscopy demonstrates that the 5-μm beads rapidly accumulate at the tips of the sharp edges, while the small 400-nm nanoparticles remain distributed throughout the channel (Fig 3A). This effect is seen across multiple sharp-edge microstructures in the device and does not occur in the absence of acoustic activation (Fig. 3B). This property of differential size-based capture of particles can be harnessed for specific detection of objects of interest, such as sEVs, in biofluids.

### Quantification of avidin-biotin binding and detection efficiency

**Fig. 4.**
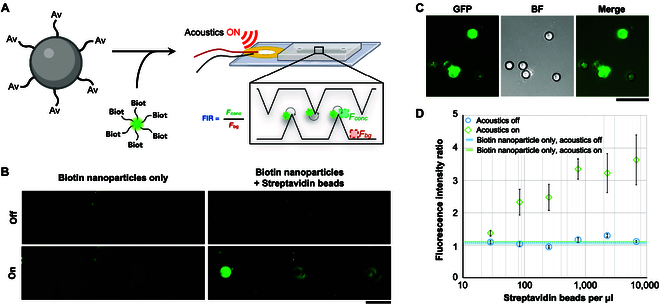
The acoustofluidic device is used to detect and isolate nanoparticle-microparticle aggregates. (A) Streptavidin-labeled microparticles are combined with fluorescent biotin-labeled nanoparticles. When the sample containing microparticle–nanoparticle aggregates is introduced into the device, the aggregates concentrate at the tips of the acoustically activated sharp-edge microstructures and can be detected by fluorescence microscopy. Signal enhancement is quantified using the FIR, calculated as the average fluorescence intensity at the sharp-edge tip region divided by that of a background region of the same area (75 μm × 75 μm). (B) In the absence of streptavidin-labeled beads, there is no interaction between the fluorescent biotin-labeled nanoparticles, no aggregate formation, and therefore minimal fluorescence detected at the tips of the sharp-edge microstructures. In the presence of streptavidin-labeled beads, streptavidin-labeled microparticle/biotin-labeled nanoparticle aggregates are expected to form. In the absence of acoustic activation, no fluorescence is seen. However, when the device is acoustically activated, the aggregates are concentrated at the tips of the sharp-edge microstructures. Scale bar, 150 μm. (C) Green fluorescent biotin-labeled 200-nm particles colocalize with nonfluorescent streptavidin-labeled 5-μm particles. Scale bar, 25 μm. (D) In the presence of an excess of fluorescent biotin-labeled nanoparticles, the fluorescence signal at the tips of the acoustically activated sharp-edge microstructures is directly proportional to the concentration of streptavidin beads. However, in the absence of streptavidin-labeled microparticles or the absence of acoustic activation, no fluorescence is seen. Data are presented as mean ± standard error (*N* = 3 technical repeats performed using the same particles and identical experimental conditions).

Next, to demonstrate the principle of bead–nanoparticle target capture and identification using the sharp-edge acoustofluidic device, we incubated 5-μm nonfluorescent streptavidin-coated polystyrene beads with 200-nm green fluorescent biotin-tagged nanoparticles (Fig. 4A). The putative bead–nanoparticle complexes were then captured and imaged in the device. When a 4-kHz, 1.31-W acoustic square wave was introduced, bead–nanoparticle complexes were captured at the tips of the sharp-edge microstructures. In contrast, in the absence of the streptavidin-coated beads, the small fluorescent biotin-tagged nanoparticles are dispersed throughout the microchannel. Prior to acoustic activation, both nanoparticles and complexes are distributed throughout the microchannel (Fig. 4B). Microscopy was used to visualize the formed complexes (Fig. 4C). The 5-μm nonfluorescent microparticles are seen on brightfield imaging, while the 200-nm nanoparticles are seen by fluorescence imaging.

To quantify the detection efficiency of bead–nanoparticle complexes within the acoustofluidic device, we incubated varying concentrations (10^1^ to 10^4^ μl^−1^) of the 5-μm streptavidin beads with an excess (2 × 10^8^ μl^−1^) of 200-nm fluorescent biotin nanoparticles. Due to the robust interaction (*K*_d_ ~ 10^−15^ M) between streptavidin and biotin [51], the detected FIR at the tip of the sharp-edge microstructures increased proportionally with the concentration of streptavidin-coated beads (Fig. 4D), indicating efficient capture of bead–nanoparticle complexes by the device. In contrast, in the absence of acoustic activation or in the absence of streptavidin-tagged beads, the FIR nears 1, as expected.

### Acoustofluidic-enabled capture and detection of sEVs via specific surface markers

**Fig. 5.**
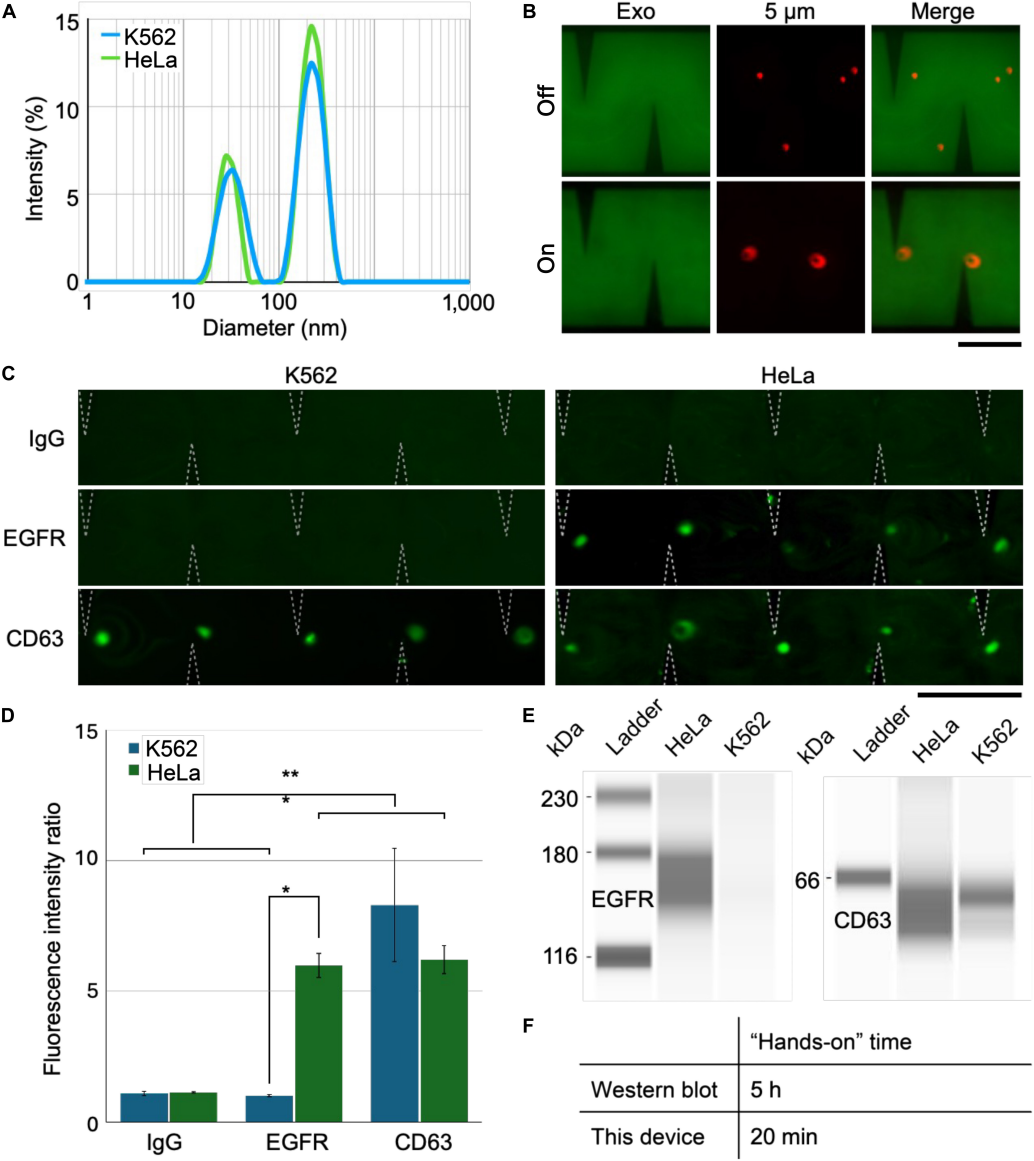
The acoustofluidic device is used to capture and detect sEVs expressing specific surface proteins. (A) Dynamic light scattering data demonstrate the nanoscale size distribution of both K562- and HeLa-derived sEVs. (B) In the absence of an acoustic activation, both 5-μm microparticles (red) and nano-sized sEVs (green) are distributed throughout the PDMS microchannel. However, under acoustic activation, the device concentrates large microparticles at the tips of the sharp-edge microstructures, while nano-sized sEVs (green) remain distributed within the channel. Scale bar, 300 μm. (C) Extracellular vesicles derived from K562 (EGFR-negative) and HeLa (EGFR-positive) cells are combined with anti-EGFR beads. In the presence of anti-EGFR beads, EGFR-positive HeLa-derived sEVs are detected at the tips of the sharp-edge microstructures within the device, while EGFR-negative K562-derived sEVs are not. Both HeLa- and K562-derived sEVs are detected in the presence of anti-CD63 beads (positive binding control), while neither is detected in the presence of anti-rat IgG beads (negative binding control). Scale bar, 400 μm. (D) In the presence of anti-EGFR beads, EGFR-positive HeLa-derived sEVs demonstrate significantly greater signal enhancement in the device compared to EGFR-negative K562-derived sEVs (*P* = 0.010). In contrast, there is no significant difference in signal enhancement in the presence of anti-rat IgG beads (*P =* 1.000) and anti-CD63 beads (*P =* 0.615). Additionally, there is a significant difference in signal enhancement between binding and nonbinding conditions (*P* < 0.001), and no significant difference between groups after binding has been accounted for (*P* = 0.460). Data are presented as mean ± standard error (*N* = 3 technical repeats performed using the same batch of cell culture and identical experimental conditions). (E) Western blotting similarly demonstrates a band at the expected size for CD63 in sEV samples derived from both K562 (EGFR-negative) and HeLa (EGFR-positive) cell lines, while only sEVs derived from HeLa cells demonstrate a band at the expected size for EGFR. (F) While specific sEV surface markers can be detected using both the acoustofluidic device and Western blotting, the “hands-on” sample processing time is 5 h or more with Western blotting, but only 20 min for the device.

To demonstrate the utility of the device for biomarker detection in biological samples, we aimed to show sEV capture and detection via specific surface markers using the acoustofluidic device. Dynamic light scattering measurements showed that the size of sEVs derived from EGFR-negative K562 cells was 159.8 nm with polydispersity index (PDI) = 0.355, while sEVs isolated from EGFR-positive HeLa cells were 202.8 nm with PDI = 0.302 (Fig. 5A). To demonstrate that large beads could be detected within a sample containing sEVs, we first introduced a mixture of 5-μm red fluorescent polystyrene beads and fluorescently stained, K562-derived sEVs into the device. The device was then activated with a 90 Vpp (1.31 W) acoustic field at 4 kHz, causing the large red fluorescent beads to be accumulate near the sharp-edge microstructures, while the much smaller green fluorescent sEVs remained distributed within the fluid channel. Meanwhile, in the absence of acoustic activation, both beads and sEVs were distributed throughout the channel (Fig. 5B). The selection of 90 Vpp was guided by stability tests and measurements of acoustic streaming velocity across various input voltages, as detailed in Fig. S3.

Next, to demonstrate specific sEV capture via surface markers, we incubated anti-EGFR-coated beads with 50 μl of fluorescently labeled K562 (EGFR-negative) and HeLa (EGFR-positive) sEVs and introduced the mixture to the sharp-edge acoustofluidic device (Fig. 5C). When these nano-sized sEVs were mixed with the antibody-coated beads, anti-EGFR bead binding of HeLa (EGFR-positive) sEVs resulted in a fluorescent signal at the tips of the sharp-edge microstructures in the acoustically activated device (Fig. 5C, right). In contrast, no signal was seen in the anti-EGFR bead/K562 (EGFR-negative) sample (Fig. 5C, left). Of note, different tips have different levels of fluorescent intensity. This may be due to the stochastic nature by which binding proliferates between the nonfluorescent 5-μm beads and the fluorescent exosomes to generate large bead–exosome complexes. To further confirm the presence of intact sEVs, we performed transmission electron microscopy (TEM) imaging on sEVs collected from the device. The observed vesicles exhibited characteristic morphology and size, as shown in Fig. S4.

To quantify signal enhancement within the device, we calculated the FIR at the tips of the sharp-edge microstructures compared to the background at the edge of the PDMS microchannel (Fig. 5D). The FIR was 6.00 ± 0.46 in the anti-EGFR bead/HeLa condition, but only 1.01 ± 0.03 in the anti-EGFR/K562 condition (*P* = 0.010). Meanwhile, both HeLa and K562 sEV samples showed fluorescent enhancement in the presence of anti-CD63 beads (positive binding control) with a FIR of 6.21 ± 0.53 and 8.30 ± 2.17, respectively (*P* = 0.615). Additionally, neither HeLa nor K562 sEVs showed fluorescent enhancement in the presence of IgG beads (negative binding control) with a FIR of 1.13 ± 0.03 and 1.10 ± 0.08, respectively (*P* = 1.000). Finally, nested ANOVA showed a significant difference in the FIR between all binding (anti-EGFR/HeLa, anti-CD63/HeLa, anti-CD63/K562) and all nonbinding (anti-EGFR/K562, IgG/HeLa, IgG/K562) groups (*P* < 0.001), and no significant difference between groups after binding versus nonbinding had been accounted for (*P* = 0.460).

Finally, to compare sEV surface protein detection using the acoustofluidic device with surface protein detection via traditional methods, we performed Western blot analysis of K562 (EGFR-negative) and HeLa (EGFR-positive) sEV samples (Fig. 5E). As expected, both samples showed a visible band at the expected size for CD63 (estimated molecular weight 59 kDa), while only HeLa-derived sEVs demonstrated a visible band at the expected size for EGFR (170 kDa). Notably, while both the acoustofluidic device and Western blot can be used for specific sEV surface protein detection, the “hands-on” sample processing and preparation time is estimated to be 5 h or more for Western blotting, but only 20 min for the acoustofluidic device (Fig. 5F).

## Discussion

SEVs are secreted by all cells into various bio-fluids, including the peripheral blood [1]. They have been used as vectors for liquid biopsy to enable minimally invasive disease detection, particularly in cancer [52,53]. However, current methods for sEV sample preparation and detection are time- and resource-intensive, typically require multiple processing steps, and often use large sample volumes [10,11,54,55]; combined, these limitations present a marked challenge for the use of sEVs in point-of-care clinical applications.

In this work, we have used a sharp-edge acoustofluidic device combined with antibody-coated microparticles for rapid and specific capture and detection of sEVs via specific surface markers in volumes as small as 50 μl. Although the dead space of the device is <5 μl, to allow optimal sample loading and maximize fluorescent readout, we have tested the device with sample volumes as low as 50 μl. The fluorescent signal is determined by the concentration of bead-bound sEVs and the volume of the microfluidic channel, independent of the total sample volume. When the device is acoustically activated, large moieties such as microparticle-bound sEVs accumulate at the tips of the sharp-edge microstructures, while small entities such as unbound sEVs remain distributed throughout the microchannel. By harnessing this size-based acoustic streaming property, we are able to capture sEVs via specific surface markers such as EGFR.

To demonstrate the functionality of the acoustofluidic device, we have used anti-EGFR beads to specifically detect sEVs derived from EGFR-positive HeLa cells within the device. The fluorescence signal enhancement for EGFR-positive sEVs at the tip of the sharp-edge microstructures within the device was 6× that of EGFR-negative sEVs. Additionally, the EGFR-negative sEVs demonstrated no signal enhancement within the acoustically activated device, as expected. Importantly, while EGFR is used in this study to demonstrate proof-of-concept for specific sEV capture, the modularity of the system allows versatile sEV detection via other surface markers of interest simply by exchanging the antibody on the surface of the capture beads.

The acoustofluidic technology described here enables highly flexible, specific, and efficient capture and detection of circulating extracellular vesicles (sEVs) from small sample volumes. Its portability, low cost, and ease of use make it an ideal tool for point-of-care detection of sEV surface markers, while its modular design allows for one-step, high-throughput capture and detection of diverse sEV populations. Additionally, the system’s ability to be easily parallelized enables simultaneous, multi-marker analysis with high precision. Future applications include the capture and detection of organ-specific sEVs from peripheral blood, enabling minimally invasive, multi-omic disease assessment beyond cancer biomarkers. Ongoing work will focus on multiplexing the device for the simultaneous detection of multiple targets, further expanding its diagnostic capabilities. By seamlessly integrating speed, accuracy, and accessibility, this platform has the potential to transform liquid biopsy, bringing precision medicine to the forefront of widespread clinical practice.

## Data Availability

The authors declare that all data supporting the findings of this study are available within the article. Further information is available from the corresponding author upon reasonable request.
