# Supplementary material for: An Acoustofluidic Device for Sample Preparation and Detection of Small Extracellular Vesicles
Source: Cyborg Bionic Syst. 2025 Jul 17;6:0319. doi: 10.34133/cbsystems.0319 (PMC12267987; doi:10.34133/cbsystems.0319)
Supplement: Supplementary 1 — Figs. S1 to S5 [file cbsystems.0319.f1.docx]

Supplementary Information

**An acoustofluidic device for sample preparation and detection of small extracellular vesicles**

Jessica F. Liu^†^, Jianping Xia^†^, Joseph Rich, Shuaiguo Zhao, Kaichun Yang, Brandon Lu, Ying Chen, Tiffany Wen Ye, and Tony Jun Huang*

* Address correspondence to: Tony Jun Huang; Email: [tony.huang@duke.edu](mailto:tony.huang@duke.edu)

This file contains Supplementary **Figs. S1-S5**

**
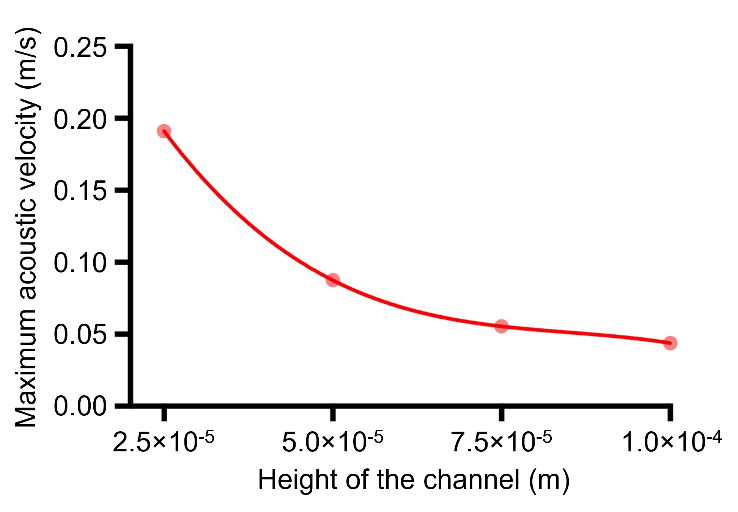
**

**Fig. S1**, Simulation of the effect of microchannel height on the maximum acoustic velocity. The results show that as the channel height increases from 25 μm to 100 μm, the maximum streaming velocity decreases significantly. A height of 50 μm was selected as a design compromise to ensure sufficient acoustic force for particle aggregation while avoiding channel blockage due to excessive confinement.


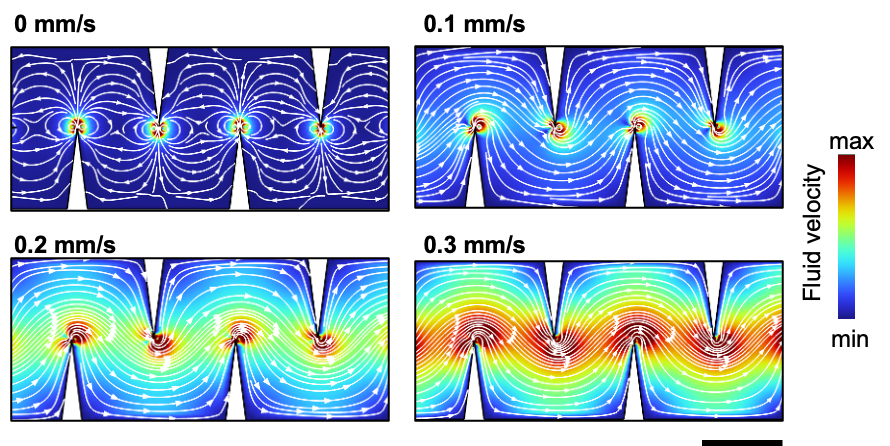


**Fig. S2**, COMSOL simulation of acoustic streaming under different background flow conditions. Acoustic streaming field without background flow shows strong, symmetric vortices centered around each sharp-edge structure. As the background flow velocity increases to 0.1 mm/s, 0.2 mm/s, and 0.3 mm/s, respectively, the streaming vortices become increasingly distorted and eventually suppressed. At 0.3 mm/s, no distinct acoustic vortex is observable, indicating that high background flow interferes with acoustic streaming and impairs particle aggregation. These results support the use of a stop-flow approach to allow acoustic streaming to dominate during operation. Scale bar = 400 µm.


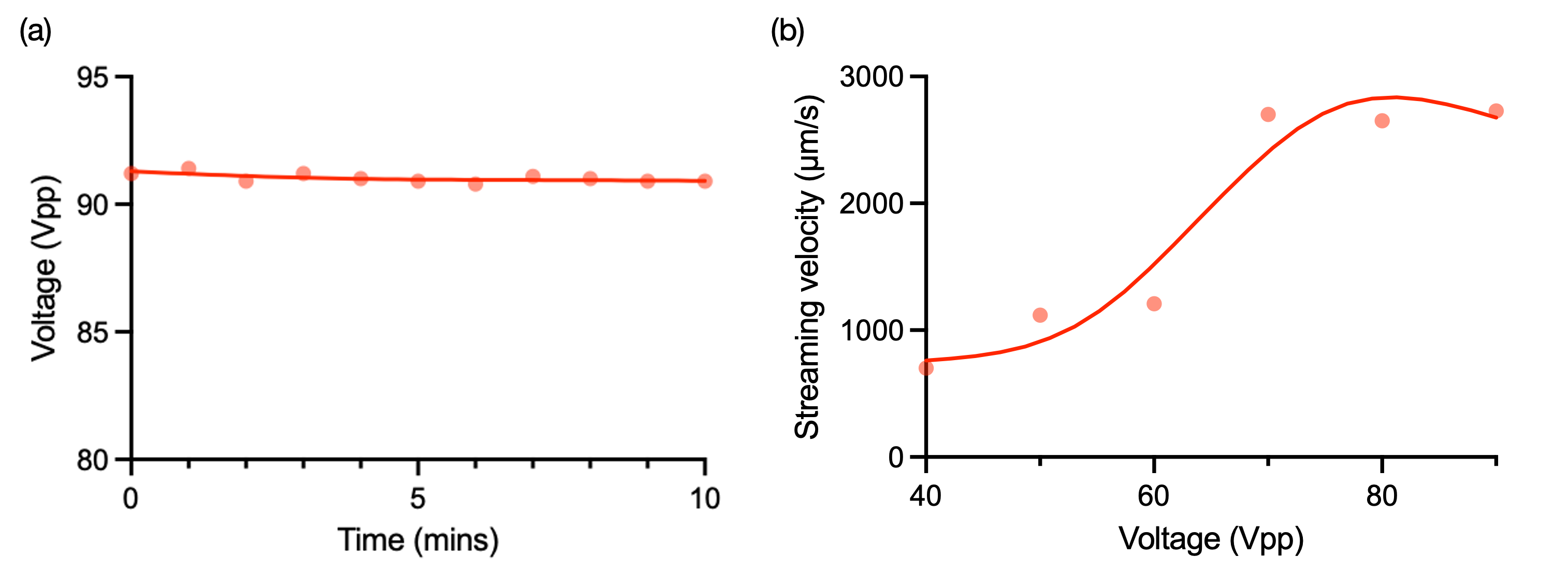


**Fig. S3**, (a) Voltage stability measurement of the acoustic transducer over 10 minutes of continuous operation at 90 Vpp. The voltage remained constant, indicating stable performance without degradation. (b) Simulated acoustic streaming velocity near the sharp-edge structure as a function of input voltage. The streaming velocity saturates near 70 Vpp, and 90 Vpp was selected to ensure consistent performance across devices.


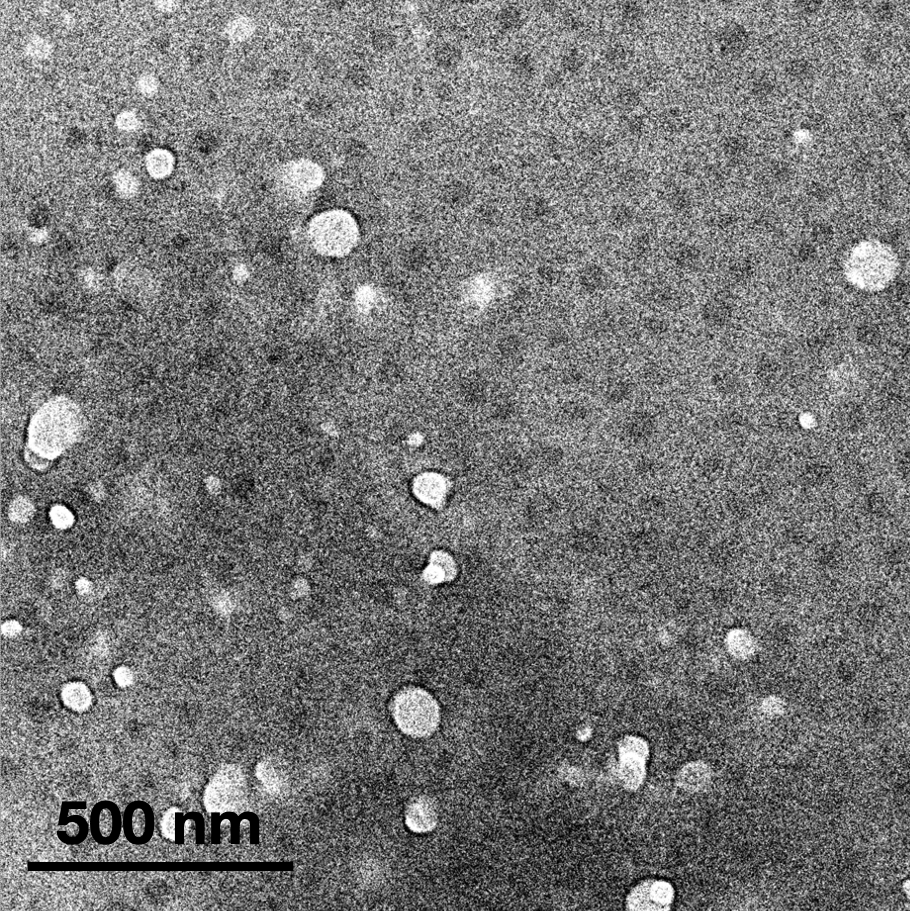


**Fig. S4,** Transmission electron microscopy (TEM) image of small extracellular vesicles (sEVs) released from bead–sEV complexes collected from the device. sEVs were released using glycine-HCl buffer (pH 2.0) and immediately neutralized with Tris buffer (pH 8.0). The observed vesicles exhibit characteristic size and morphology, confirming the feasibility of downstream sEV recovery and analysis. Scale bar: 500 nm.


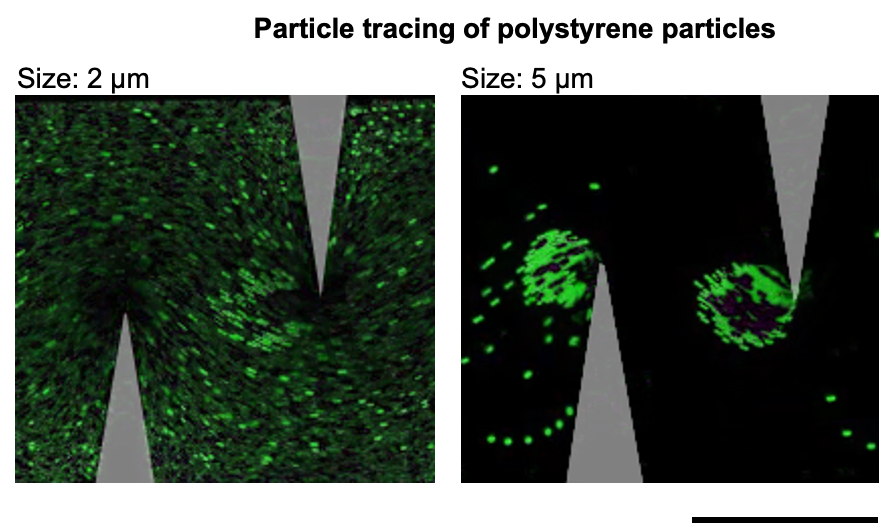


**Fig. S5.** Comparison of microparticle aggregation using 2 μm (left) and 5 μm (right) beads under identical acoustic actuation conditions. The 2 μm particles exhibited weak and dispersed fluorescence signals, indicating poor aggregation. In contrast, 5 μm particles formed stable and well-defined clusters around the sharp-edge structures, demonstrating more effective acoustic manipulation. The enhanced aggregation with 5 μm particles is attributed to the size-dependent increase in acoustic radiation and drag forces. Additionally, 5 μm particles coated with CD63 were commercially available, enabling reliable and reproducible sEV capture in the system. Scale bar = 400 µm.
